# Supplementary material for: Phytate and Microbial Suspension Amendments Increased Soybean Growth and Shifted Microbial Community Structure
Source: Microorganisms. 2021 Aug 25;9(9):1803. doi: 10.3390/microorganisms9091803 (PMC8471086; doi:10.3390/microorganisms9091803)
Supplement: Supplementary file 1 [file microorganisms-09-01803-s001.zip › microorganisms-1322016-supplementary.pdf]

Supplementary Information

- ***Supplementary Figures S1-S5 (pages 1-5)***
- ***Supplementary Tables S1-S10 (pages 6-25)***

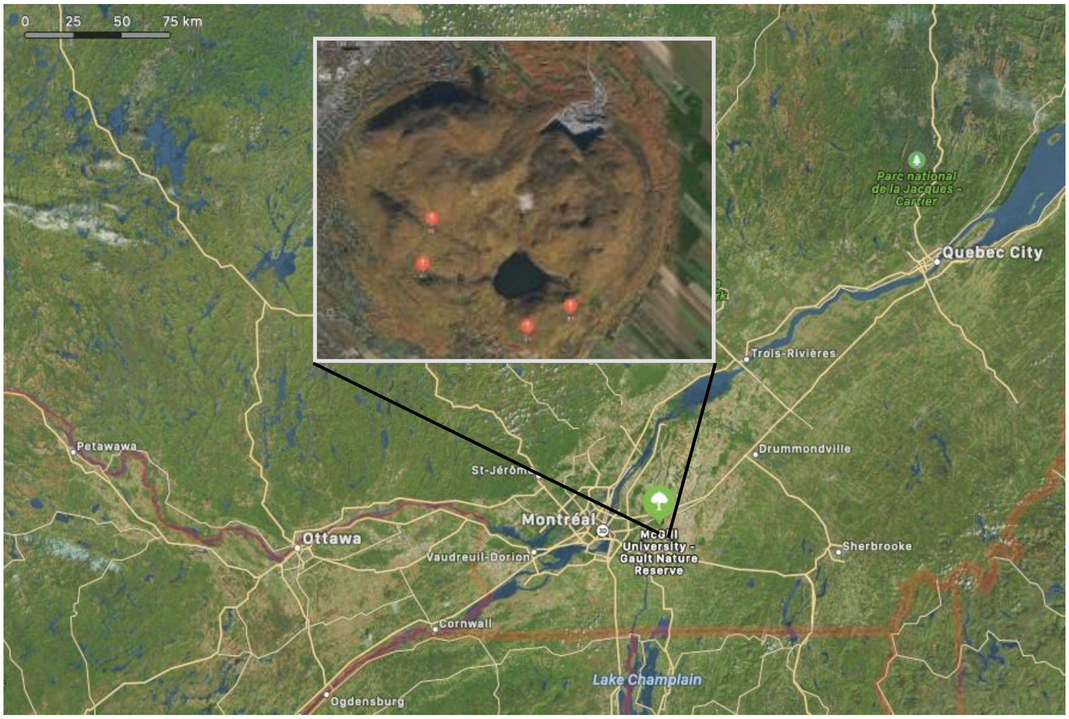

Figure S1. Map of Gault Nature Reserve. Sampling sites (S1-S4) for soil to prepare microbial inoculum used in the experiment.

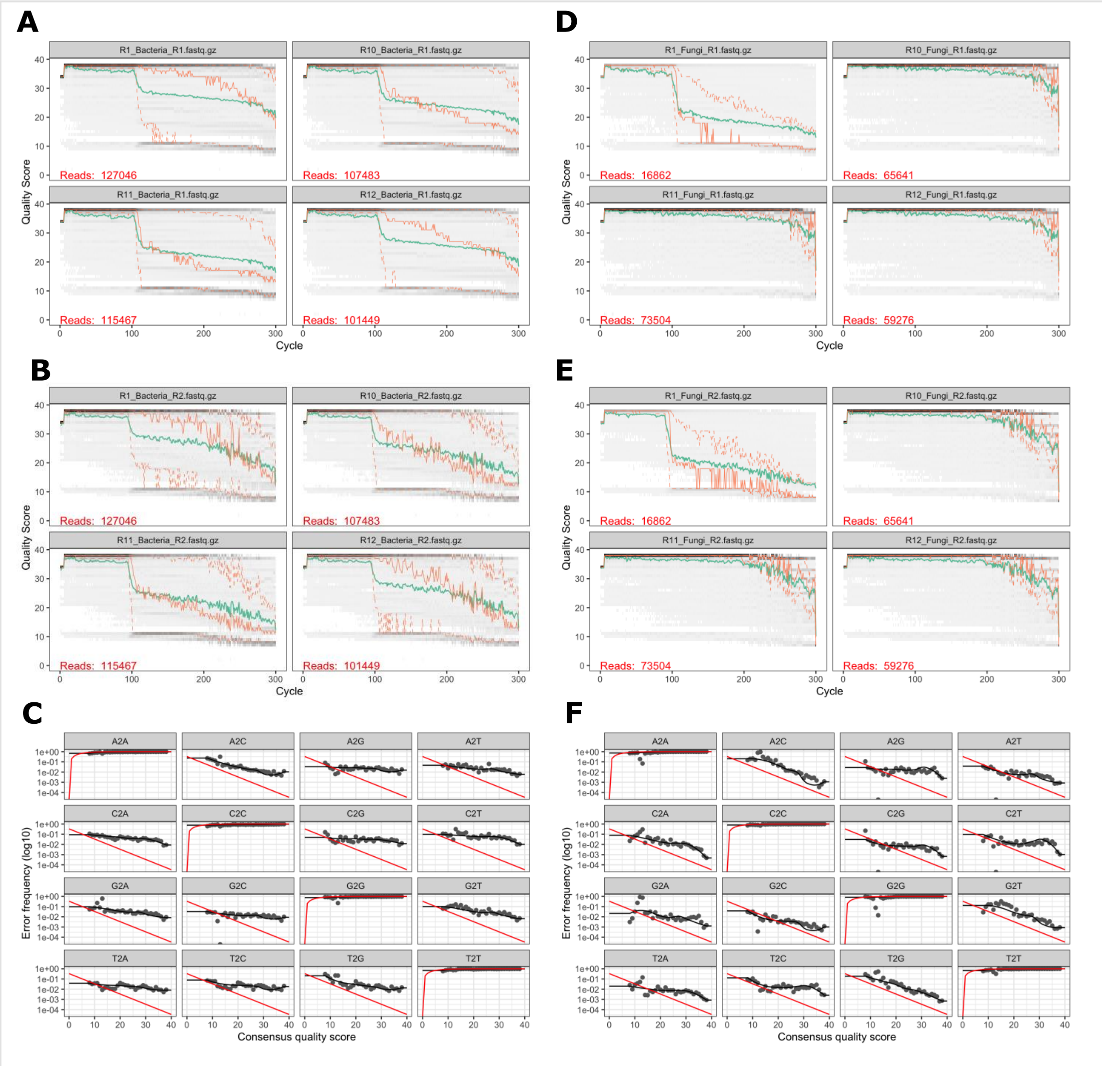

Figure S2. Quality profiles and error rates of raw reads. Quality profiles for 16sRNA forward reads (A), reverse reads (B), and ITS forward (D) and reverse reads (E). The green line denotes the mean quality score, the orange line denotes the quartiles, and the gray line denotes the frequency of each quality score at each base position. The error rates for each possible transposition are shown for 16s RNA (C) and ITS (F) reads.

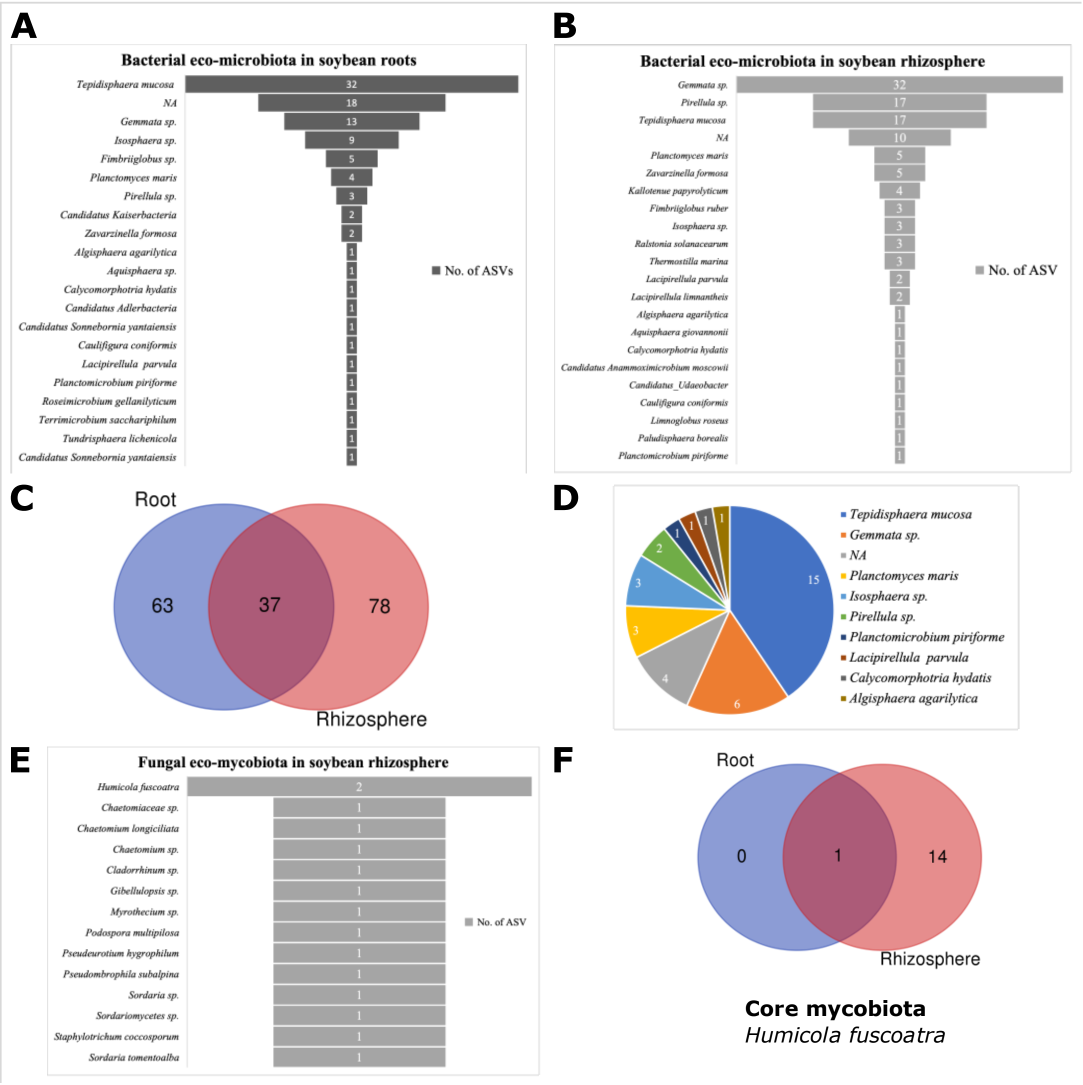

Figure S3. Eco and core microbiota in soybean microbiome. Bacterial eco-microbiota in root (A) and rhizosphere (B) biotopes. The Venn diagram shows the number of bacterial ASVs that were unique or shared in root and rhizosphere biotopes (C). The pie chart shows bacterial core microbiota (D). (E) Fungal eco-mycobiota in rhizosphere soil. (F) The Venn diagram shows the number of ASVs found unique or shared in root and rhizosphere biotopes. *Humicola fuscoatra* was the only core fungal microbe.

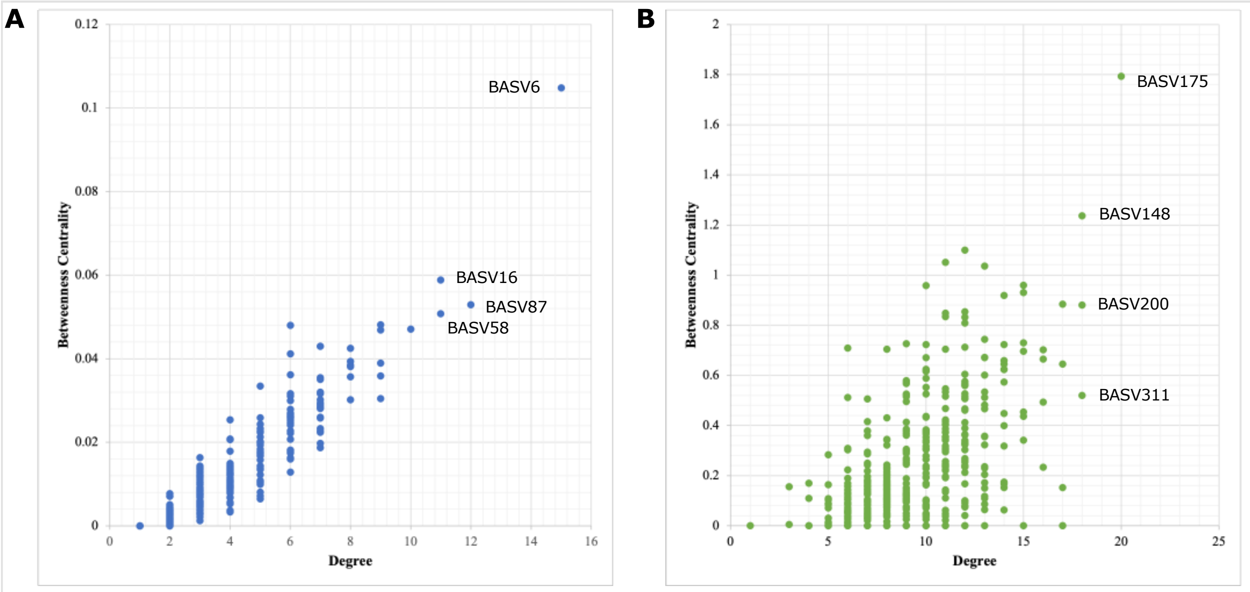

Figure S4. Scatter plot of the node degree versus betweenness centrality of ASVs in the root (A) and rhizosphere (B) microbiota.

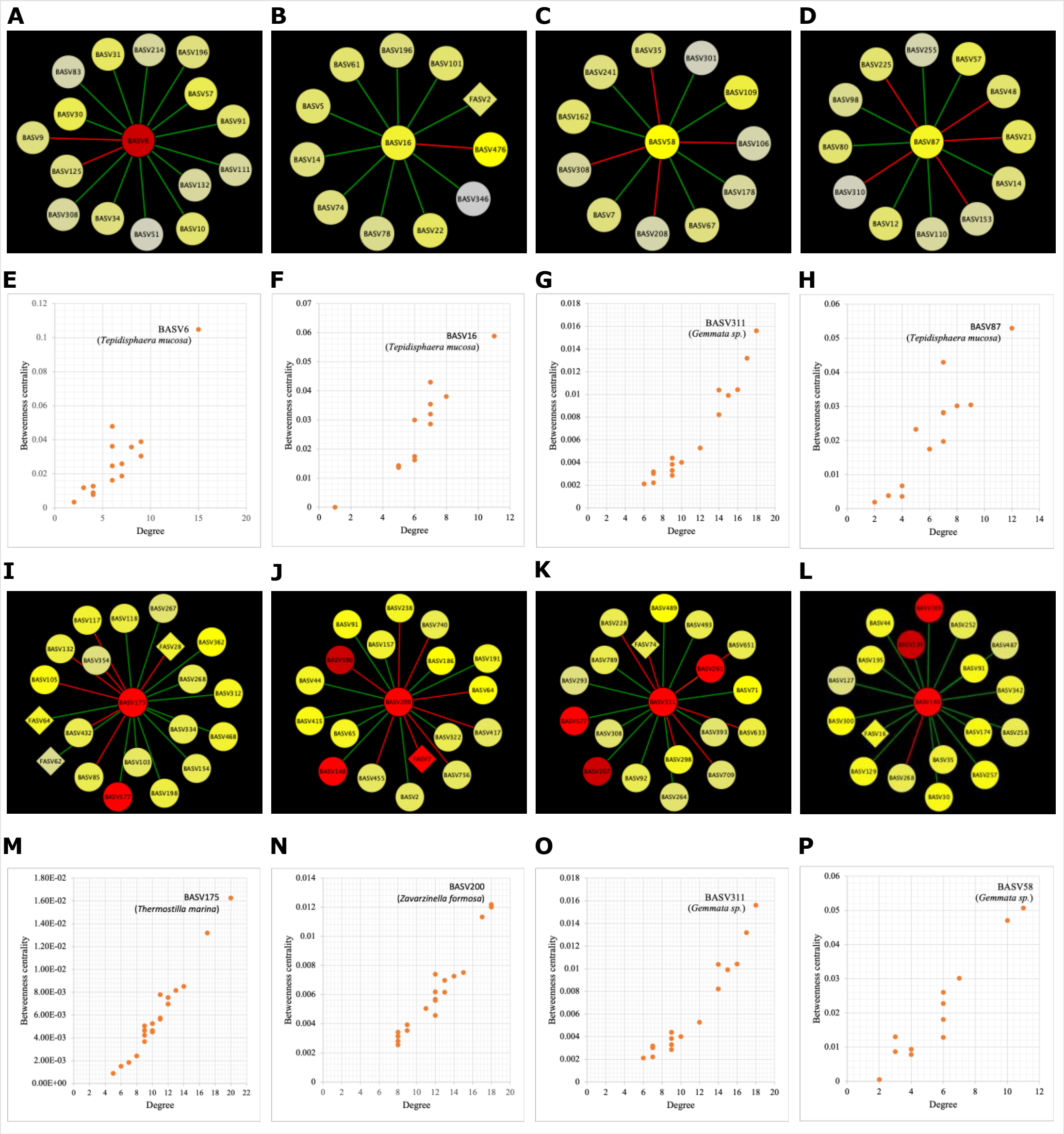

Figure S5. Subnetwork of the hub taxa. The co-occurrence of the hub taxa in root biotope (A-D), and scatter plot of the node degree versus betweenness centrality of ASVs in the corresponding subnetworks (E-H). The co-occurrence network of the hub taxa in rhizosphere biotope (I-L), and scatter plot representing the node degree versus betweenness centrality of ASVs in the corresponding subnetworks (M-P). Green link represents a positive interaction while red link represents a negative interaction. Nodes are coloured according to the relative abundance of the corresponding ASVs, and node shapes represent bacterial (circular) and fungal (rhombus) ASVs.

Supplementary Tables S1-S10

Table S1. Phylum identified in the bacterial communities.

Phylum	sum of relative abundance	Sum of total abundance
Planctobacteria	0.761581306	1529236.222
Patescibacteria	0.073669631	147926.7778
Verrucomicrobiota	0.037807465	75916.44444
Chloroflexi	0.026531594	53274.77778
Proteobacteria	0.01349386	27095.33333
Actinobacteria	0.004860895	9760.555556
Acidobacteria	0.00384395	7718.555556
Firmicutes	0.001811499	3637.444444
NA	0.001131046	2271.111111
Tenericutes	0.001098232	2205.222222
Armatimonadota	0.001002835	2013.666667
Abditibacteriota	0.000924978	1857.333333
Myxococcota	0.000465201	934.1111111
Gemmatimonadota	0.000386514	776.1111111
Deinococcota	0.00024209	486.1111111
Bacteroidota	0.000224383	450.5555556
Methylomirabilota	0.000158811	318.8888889
Bdellovibrionota	0.000114377	229.6666667
Cyanobacteria	8.21723E-05	165
Hydrogenedentes	7.56982E-05	152
Nitrospirae	6.77299E-05	136
Desulfobacterota	3.04342E-05	61.11111111
Dependentiae	2.98809E-05	60
Elusimicrobiota	2.68928E-05	54
Gemmatimonadetes	1.69325E-05	34
Latescibacterota	1.54384E-05	31
Calditrichaeota	1.04583E-05	21
Spirochaetota	5.97617E-06	12
Kiritimatiellaeota	4.98014E-06	10
Dadabacteria	4.98014E-06	10
Sumerlaeota	3.98411E-06	8
Thermodesulfobacteria	3.4861E-06	7
Saccharibacteria	3.32009E-06	6.666666667
Proteobacteria	2.76675E-06	5.555555556
Elusimicrobia	2.49007E-06	5
Armatimonadetes	1.99206E-06	4
WS2	1.99206E-06	4
NB1-j	1.99206E-06	4
WPS-2	9.96028E-07	2

Table S2. Orders identified in the bacterial communities.

Order	sum of relative abundance	Sum of total abundance
Tepidisphaerales	0.255766575	513572.8889
Gemmatales	0.227298038	456408.7778
Pirellulales	0.10903406	218937.6667
Isosphaerales	0.091884721	184502.2222
Candidatus_Kaiserbacteria	0.055325335	111091.8889
Planctomycetales	0.052110764	104637.1111
Chthoniobacterales	0.027142215	54500.88889
Phycisphaerales	0.017434591	35008.22222
Burkholderiales	0.013807443	27725
Kallotenuales	0.009609515	19295.66667
NA	0.007067264	14190.88889
Candidatus_Adlerbacteria	0.005967538	11982.66667
Verrucomicrobiales	0.004891052	9821.111111
Candidatus Brocadiales	0.003938628	7908.666667
Chloroflexales	0.003514541	7057.111111
Saccharimonadales	0.003388931	6804.888889
Pedosphaerales	0.003162999	6351.222222
Thermomicrobiales	0.002477178	4974.111111
Chlamydiales	0.001877403	3769.777778
Rhizobiales	0.001761808	3537.666667
Dehalococcoidales	0.001740394	3494.666667
Sphaerobacterales	0.001716157	3446
Candidatus_Zambryskibacteria	0.001710236	3434.111111
Micrococcales	0.001691643	3396.777778
Vicinamibacterales	0.001562381	3137.222222
Candidatus_Nomurabacteria	0.001508208	3028.444444
Candidatus_Azambacteria	0.001344251	2699.222222
Mycoplasmatales	0.001098232	2205.222222
Pla1_lineage	0.001016779	2041.666667
Fimbriimonadales	0.001003831	2015.666667
Candidatus	0.000959452	1926.555556
CSphingomonadales	0.000928962	1865.333333
Abditibacteriales	0.000924978	1857.333333
Bacillales	0.000726769	1459.333333
Xanthomonadales	0.000715259	1436.222222
Streptomycetales	0.000631537	1268.111111
Caulobacterales	0.000544606	1093.555556
Caldilineales	0.00045856	920.7777778
Propionibacteriales	0.000449596	902.7777778
Tepidiformales	0.00044196	887.4444444
Subgroup_7	0.000418166	839.6666667

Blastocatellales	0.000407708	818.6666667
Opitutales	0.000404055	811.3333333
Candidatus_Moranbacteria	0.000379542	762.1111111
Pyrinomonadales	0.00037672	756.4444444
Pseudomonadales	0.000371519	746
Gemmatimonadales	0.000351155	705.1111111
Clostridiales	0.000329021	660.6666667
Gaiellales	0.000305393	613.2222222
Acidobacteriales	0.000300192	602.7777778
Frankiales	0.000280216	562.6666667
Solirubrobacterales	0.000276841	555.8888889
Micromonosporales	0.000250224	502.4444444
Deinococcales	0.00024209	486.1111111
Pseudonocardiales	0.000235007	471.8888889
Candidatus_Lloydbacteria	0.000218739	439.2222222
Planctomycetales	0.000203356	408.3333333
CCM11a	0.000201806	405.2222222
Blastocatellales	0.000192565	386.6666667
Microtrichales	0.000190463	382.4444444
Haliangiales	0.000181997	365.4444444
Limnochordia_or	0.000170708	342.7777778
Rokubacteriales	0.000158811	318.8888889
Ktedonobacterales	0.000156155	313.5555556
Clostridiales	0.000155878	313
Anaerolineales	0.000154716	310.6666667
Natranaerobiales	0.000144701	290.5555556
Polyangiales	0.000139555	280.2222222
Holophagales	0.000136954	275
SBR1031	0.000130037	261.1111111
Corynebacteriales	0.000128322	257.6666667
Verrucomicrobiales	0.000127436	255.8888889
Myxococcales	0.000115539	232
PaeniBacillales	0.000111666	224.2222222
Solibacterales	0.000110282	221.4444444
Chitinophagales	0.000107516	215.8888889
Omnitrophales	0.000106796	214.4444444
Actinomycetales	9.62274E-05	193.2222222
Thermoanaerobaculales	9.52867E-05	191.3333333
Bdellovibrionales	9.27966E-05	186.3333333
RBG-13-54-9	8.70418E-05	174.7777778
Anaerolineales	8.12316E-05	163.1111111
Gammaproteobacteria_Incertae_Sedis	7.96269E-05	159.8888889

Candidatus_Campbellbacteria	7.76349E-05	155.8888889
Hydrogenedentiales	7.56982E-05	152
GWA2-38-13b	7.33188E-05	147.2222222
Desulfobacterales	7.23227E-05	145.2222222
Subgroup_17	7.105E-05	142.6666667
Methylococcales	6.66786E-05	133.8888889
Streptosporangiales	6.22518E-05	125
Limnochordales	6.18091E-05	124.1111111
Acidimicrobiales	6.17538E-05	124
C0119	5.78803E-05	116.2222222
Flavobacteriales	5.7825E-05	116.1111111
Methylacidiphilales	5.7659E-05	115.7777778
Micropepsales	5.68843E-05	114.2222222
Nitrospirales	5.47816E-05	110
Candidatus_Spechtbacteria	5.47816E-05	110
Rhodospirillales	5.26235E-05	105.6666667
Obscuribacterales	4.99121E-05	100.2222222
Vicinamibacteraceae	4.93587E-05	99.11111111
Dongiales	4.7754E-05	95.88888889
Candidatus_Uhrbacteria	4.65367E-05	93.44444444
Desulfuromonadales	4.56513E-05	91.66666667
Bacteriovoracales	4.19992E-05	84.33333333
IMCC26256	3.98965E-05	80.11111111
Candidatus_Magasanikbacteria	3.55803E-05	71.44444445
Longimicrobiales	3.23709E-05	65
S-BQ2-57_soil_group	3.20389E-05	64.33333333
Acetobacterales	3.03789E-05	61
Kineosporiales	3.03789E-05	61
Sphingobacteriales	3.01022E-05	60.44444445
Babeliales	2.98809E-05	60
Geobacterales	2.94382E-05	59.11111111
Azospirillales	2.94382E-05	59.11111111
Steroidobacterales	2.93828E-05	59
DS-100	2.80548E-05	56.33333333
Bryobacterales	2.77781E-05	55.77777778
Cytophagales	2.69481E-05	54.11111111
Subgroup_2	2.58967E-05	52
Brocadiales	2.4624E-05	49.44444444
Synechococcales	2.09719E-05	42.11111111
Desulfobacterales	1.98099E-05	39.77777778
Dehalococcoides	1.90905E-05	38.33333333
Diplorickettsiales	1.89799E-05	38.11111111

Syntrophobacterales	1.82605E-05	36.66666667
Thermoanaerobacterales	1.73198E-05	34.77777778
Desulfovibrionales	1.64345E-05	33
Sedimentisphaerales	1.56598E-05	31.44444444
Lachnospirales	1.49404E-05	30
mle1-8	1.48298E-05	29.77777778
Catenulisporales	1.44977E-05	29.11111111
AlicycloBacillales	1.39444E-05	28
Peptostreptococcales-Tissierellales	1.39444E-05	28
Oligoflexales	1.31144E-05	26.33333333
Rhodobacterales	1.3059E-05	26.22222222
Nitrospinales	1.29484E-05	26
Elsterales	1.14543E-05	23
Reyranellales	1.06796E-05	21.44444444
Calditrichales	1.04583E-05	21
Vibrionales	9.73894E-06	19.55555556
Clostridia_or	8.46624E-06	17
Rickettsiales	8.46624E-06	17
Chromatiales	8.46624E-06	17
Acidithiobacillales	8.46624E-06	17
Leptolyngbyales	8.30024E-06	16.66666667
Thermoactinomycetales	7.96823E-06	16
Salinisphaerales	7.96823E-06	16
Thermobaculales	7.47021E-06	15
Tistrellales	7.47021E-06	15
Defluviicoccales	6.9722E-06	14
Nannocystales	6.69552E-06	13.44444444
Rubrobacterales	6.36351E-06	12.77777778
S-70	6.30818E-06	12.66666667
Spirochaetales	5.97617E-06	12
Candidatus_Jorgensenbacteria	5.97617E-06	12
o_clostridiales	5.47816E-06	11
Veillonellales-Selenomonadales	5.47816E-06	11
CCD24	4.98014E-06	10
Dadabacteriales	4.98014E-06	10
Kiritimatiellales	4.98014E-06	10
mle1-27	4.98014E-06	10
Erysipelotrichales	4.98014E-06	10
Candidatus_Vogelbacteria	4.48213E-06	9
0319-6G20	3.98411E-06	8
Rhodocyclales	3.98411E-06	8
Sumerlaeales	3.98411E-06	8

Candidatus_Falkowbacteria	3.98411E-06	8
Thermodesulfobacteriales	3.4861E-06	7
Cellvibrionales	3.4861E-06	7
Saccharibacteria	3.32009E-06	6.666666667
Bifidobacteriales	3.32009E-06	6.666666667
Legionellales	2.98809E-06	6
Nevskiales	2.98809E-06	6
Desulfovibrionales	2.76675E-06	5.555555556
Coxiellales	2.76675E-06	5.555555556
Lineage_IV	2.49007E-06	5
Enterobacterales	2.49007E-06	5
Thermosediminibacterales	2.49007E-06	5
Sphingomonadales	2.49007E-06	5
Elusimicrobiales	2.49007E-06	5
Desulfarculales	2.2134E-06	4.444444444
Desulfitobacteriales	1.99206E-06	4
Candidatus_Giovannonibacteria	1.99206E-06	4
Gastranaerophilales	1.99206E-06	4
CHAB-XI-27	1.49404E-06	3
PLTA13	1.49404E-06	3
Syntrophomonadales	1.49404E-06	3
Thermacetogeniales	1.49404E-06	3
BreviBacillales	9.96028E-07	2
AKIW659	9.96028E-07	2
Armatimonadales	9.96028E-07	2
AT-s2-59	9.96028E-07	2
Thermincolales	9.96028E-07	2
Desulfobulbales	9.96028E-07	2
Oscillospirales	9.96028E-07	2

Table S3. Bacterial indicator species in roots and rhizosphere in response to microbial inoculum and phytate.

A. Bacterial indicator species in roots in response to microbial inoculum and phytate

ASVs	p. value	Phylum	Class	Order	Family	Genus	Species
BASV738	0.018 *	Planctobacteria	Phycisphaerae	Tepidisphaerales	Tepidisphaeraceae	*Tepidisphaera*	*T. mucosa*
BAS766	0.025 *	Planctobacteria	Planctomycetes	Pirellulales	Pirellulaceae	*Candidatus*	*C. Anammoximicrobium moscowii*

B. Bacterial indicator species in rhizosphere in response to microbial inoculum and phytate

ASVs	p. value	Phylum	Class	Order	Family	Genus	Species
BASV1092	0.028 *	Planctobacteria	Planctomycetes	Pirellulales	Pirellulaceae	*Pirellula*	*Pirellula sp.*

Table S4. Phylum identified in the fungal communities.

Phylum	sum of relative abundance	Sum of total abundance
Ascomycota	0.752207572	1510414
Basidiomycota	0.022838432	45859
NA	0.014032545	28177
Mucoromycota	0.009352706	18780
Chytridiomycota	0.003985109	8002
Blastocladiomycota	2.98809E-06	6

Table S5. Orders identified in the fungal communities.

Order	sum of relative abundance	Sum of total abundance
Sordariales	0.461950472	927585
NA	0.097319439	195415
Hypocreales	0.038086132	76476
Glomerellales	0.032927203	66117
Pleosporales	0.030766319	61778
Pezizales	0.028114394	56453
Orbiliales	0.027313089	54844
Agaricales	0.011032508	22153
Myrmecridiales	0.007411447	14882
Helotiales	0.00633723	12725
Eurotiales	0.006292907	12636
Mortierellales	0.005224169	10490
Onygenales	0.004623564	9284
Microascales	0.0044388	8913
Annulatascales	0.003872558	7776
Xylariales	0.003684309	7398
Filobasidiales	0.003463689	6955
Saccharomycetales	0.00282623	5675
Coniochaetales	0.002820752	5664
Chaetosphaeriales	0.002618558	5258

Chaetothyriales	0.002430309	4880
Sebacinales	0.002313774	4646
Glomerales	0.001879505	3774
Venturiales	0.001817254	3649
Cantharellales	0.001458683	2929
Rhizophlyctidales	0.001391452	2794
Spizellomycetales	0.001173321	2356
Paraglomerales	0.00090041	1808
Auriculariales	0.000851604	1710
Trichosporonales	0.00084264	1692
Cladosporiales	0.000729591	1465
Tremellales	0.000599111	1203
Pyxidiophorales	0.000590645	1186
Russulales	0.000578692	1162
Diversisporales	0.000513951	1032
Polyporales	0.000508472	1021
Cystofilobasidiales	0.000344128	691
Geminibasidiales	0.000271418	545
Archaeosporales	0.000237553	477
Thelebolales	0.000202692	407
Mucorales	0.000177293	356
Corticiales	0.000169823	341
Muyocopronales	0.00014293	287
Rhizophydiales	0.000109563	220
Lobulomycetales	0.000100101	201
Pleurotheciales	8.91445E-05	179
Thelephorales	6.82279E-05	137
Polychytriales	6.77299E-05	136
Holtermanniales	6.02597E-05	121
Platygloeales	5.87657E-05	118
Sporidiobolales	5.12955E-05	103
Minutisphaerales	4.98014E-05	100
Mycosphaerellales	3.73511E-05	75
Pisorisporiales	3.33669E-05	67
Eremomycetales	3.28689E-05	66
Kirschsteiniotheliales	3.03789E-05	61
Lulworthiales	2.93828E-05	59
Ophiostomatales	2.68928E-05	54
Tubeufiales	2.63948E-05	53
Chytridiales	2.58967E-05	52
Spiculogloeales	2.53987E-05	51
Leucosporidiales	2.04186E-05	41

Urocystidales	1.74305E-05	35
Malasseziales	1.69325E-05	34
Caliciales	1.54384E-05	31
Togniniales	1.49404E-05	30
Lecanorales	1.44424E-05	29
Lecideales	1.39444E-05	28
Atheliales	1.09563E-05	22
Pythiales	1.04583E-05	21
Savoryellales	1.04583E-05	21
Boletales	9.46227E-06	19
Trichosphaeriales	9.46227E-06	19
Diaporthales	8.96426E-06	18
Endogonales	8.96426E-06	18
Trechisporales	8.96426E-06	18
Dothideales	7.47021E-06	15
Kriegeriales	6.9722E-06	14
Gomphales	6.47418E-06	13
Hymenochaetales	4.98014E-06	10
Rhytismatales	4.98014E-06	10
Conioscyphales	4.48213E-06	9
Erysiphales	3.4861E-06	7
Xenospadicoidales	2.98809E-06	6
Phaeothecales	2.49007E-06	5
Entorrhizales	1.99206E-06	4
Erythrobasidiales	1.99206E-06	4
Geastrales	1.99206E-06	4
Physodermatales	1.99206E-06	4
Botryosphaeriales	1.49404E-06	3
Cystobasidiales	1.49404E-06	3
Blastocladiales	9.96028E-07	2

Table S6. Fungal indicator species in roots and rhizosphere in response to microbial inoculum and phytate.

A. fungal indicator species in roots in response to microbial inoculum and phytate (M1P1)

ASVs	p. value	Phylum	Class	Order	Family	Genus	Species
FASV241	0.038	Basidiomycota	Agaricomycetes	Sebacinales	Sebacinaceae	Sebacina	Sebacina sp.
FASV46	0.032	Ascomycota	Sordariomycetes	Sordariales	Chaetomiaceae	Chaetomium	Chaetomium grande

B. fungal indicator species in rhizosphere in response to microbial inoculum and phytate (M1P1)

ASVs	p. value	Phylum	Class	Order	Family	Genus	Species
FASV513	0.007 **	NA	NA	NA	NA	NA	NA
FASV193	0.015 *	Ascomycota	Sordariomycetes	Sordariales	Chaetomiaceae	Dichotomopilus	Dichotomopilus sp.
FASV790	0.015 *	Ascomycota	Sordariomycetes	Sordariales	Chaetomiaceae	Chaetomium	Chaetomium sp.
FASV2038	0.044 *	Basidiomycota	Agaricomycetes	Agaricales	Entolomataceae	Entoloma	Entoloma sp.
FASV1066	0.024 *	Ascomycota	Sordariomycetes	Coniochaetales	Coniochaetaceae	NA	Coniochaetaceae sp.
FASV209	0.007 **	Ascomycota	Dothideomycetes	Pleosporales	Sporormiaceae	Preussia	Preussia terricola
FASV98	0.042 *	Dothideomycetes	Pleosporales	Sporormiaceae	Preussia	Preussia	Preussia sp.
FASV1111	0.039 *	Ascomycota	Sordariomycetes	Microascales	Halosphaeriaceae	NA	Halosphaeriaceae sp.
FASV693	0.041 *	Chytridiomycota	Chytridiomycetes	Spizellomycetales	Spizellomycetaceae	Spizellomyces	Spizellomyces sp.
FASV1203	0.017 *	Mucoromycota	Glomeromycetes	Glomerales	Glomeraceae	Funneliformis	Funneliformis mosseae
FASV1098	0.047 *	Ascomycota	Dothideomycetes	Pleosporales	Corynesporascaceae	Corynespora	Corynespora sp.
FASV184	0.048 *	NA	NA	NA	NA	NA	NA

Table S7. Bacterial eco-microbiota in soybean root.

ASVs	Phylum	Class	Order	Family	Genus	Species
ASV1	Planctobacteria	Phycisphaerae	Tepidisphaerales	Tepidisphaeraceae	Tepidisphaera	Tepidisphaera mucosa
ASV2	Planctobacteria	Phycisphaerae	Tepidisphaerales	Tepidisphaeraceae	Tepidisphaera	Tepidisphaera mucosa
ASV4	Patescibacteria	Parcubacteria	Candidatus_Kaiserbacteria	NA	Candidatus Kaiserbacteria	Candidatus Kaiserbacteria

ASV5	Planctobacteria	Phycisphaerae	Tepidisphaerales	Tepidisphaeraceae	Tepidisphaera	Tepidisphaera mucosa
ASV6	Planctobacteria	Phycisphaerae	Tepidisphaerales	Tepidisphaeraceae	Tepidisphaera	Tepidisphaera mucosa
ASV7	Planctobacteria	Planctomycetes	Isosphaerales	Isosphaeraceae	Isosphaera	Isosphaera sp.
ASV8	Planctobacteria	Planctomycetes	Gemmatales	Gemmataceae	Gemmata	Gemmata sp.
ASV9	Planctobacteria	Planctomycetes	Gemmatales	Gemmataceae	Fimbriiglobus	Fimbriiglobus sp.
ASV10	Planctobacteria	Phycisphaerae	Tepidisphaerales	Tepidisphaeraceae	Tepidisphaera	Tepidisphaera mucosa
ASV11	Planctobacteria	Planctomycetes	Isosphaerales	Isosphaeraceae	Isosphaera	Isosphaera sp.
ASV12	Planctobacteria	Phycisphaerae	Tepidisphaerales	Tepidisphaeraceae	Tepidisphaera	Tepidisphaera mucosa
ASV13	Planctobacteria	Planctomycetes	Isosphaerales	Isosphaeraceae	Isosphaera	Isosphaera sp.
ASV14	Planctobacteria	Phycisphaerae	Tepidisphaerales	Tepidisphaeraceae	Tepidisphaera	Tepidisphaera mucosa
ASV15	Planctobacteria	Phycisphaerae	Phycisphaerales	Phycisphaeraceae	Algisphaera	Algisphaera agarilytica
ASV16	Planctobacteria	Phycisphaerae	Tepidisphaerales	Tepidisphaeraceae	Tepidisphaera	Tepidisphaera mucosa
ASV17	Planctobacteria	Planctomycetes	Isosphaerales	Isosphaeraceae	Isosphaera	Isosphaera sp.
ASV18	Verrucomicrobiota	Verrucomicrobiae	Chthoniobacterales	Terrimicrobiaceae	Terrimicrobium	Terrimicrobium sacchariphilum
ASV21	Planctobacteria	Planctomycetes	Pirellulales	Pirellulaceae	Lacipirellula	Lacipirellula parvula
ASV22	Planctobacteria	Phycisphaerae	Tepidisphaerales	Tepidisphaeraceae	Tepidisphaera	Tepidisphaera mucosa
ASV23	Planctobacteria	Planctomycetes	Gemmatales	Gemmataceae	Gemmata	Gemmata sp.
ASV24	Planctobacteria	Planctomycetes	Gemmatales	Gemmataceae	Zavarzinella	Zavarzinella formosa
ASV25	Planctobacteria	Phycisphaerae	Tepidisphaerales	Tepidisphaeraceae	Tepidisphaera	Tepidisphaera mucosa
ASV28	Patescibacteria	Parcubacteria	Candidatus_Sonnebornia	NA	Candidatus	Candidatus Sonnebornia yantaiensis
ASV29	Planctobacteria	Phycisphaerae	Tepidisphaerales	Tepidisphaeraceae	Tepidisphaera	Tepidisphaera mucosa
ASV30	Planctobacteria	Phycisphaerae	Tepidisphaerales	Tepidisphaeraceae	Tepidisphaera	Tepidisphaera mucosa
ASV31	Planctobacteria	Phycisphaerae	Tepidisphaerales	Tepidisphaeraceae	Tepidisphaera	Tepidisphaera mucosa
ASV32	Planctobacteria	Planctomycetes	Planctomycetales	Planctomycetaceae	Planctomyces	Planctomyces maris

ASV33	Planctobacteria	Phycisphaerae	Tepidisphaerales	Tepidisphaeraceae	Tepidisphaera	Tepidisphaera mucosa
ASV34	Planctobacteria	Phycisphaerae	Tepidisphaerales	Tepidisphaeraceae	Tepidisphaera	Tepidisphaera mucosa
ASV35	Planctobacteria	Phycisphaerae	Tepidisphaerales	Tepidisphaeraceae	Tepidisphaera	Tepidisphaera mucosa
ASV36	Planctobacteria	Planctomycetes	Gemmatales	Gemmataceae	Gemmata	Gemmata sp.
ASV37	Planctobacteria	Planctomycetes	Gemmatales	Gemmataceae	Gemmata	Gemmata sp.
ASV38	Planctobacteria	Planctomycetes	Gemmatales	Gemmataceae	Gemmata	Gemmata sp.
ASV40	Planctobacteria	Planctomycetes	Gemmatales	Gemmataceae	Gemmata	Gemmata sp.
ASV41	Planctobacteria	Planctomycetes	Gemmatales	Gemmataceae	Fimbriiglobus	Fimbriiglobus sp.
ASV42	Planctobacteria	Planctomycetes	Isosphaerales	Isosphaeraceae	Tundrisphaera	Tundrisphaera lichenicola
ASV43	Parcubacteria	Candidatus_Sonnebornia	NA	Candidatus	Candidatus Sonnebornia yantaiensis	
ASV45	Planctobacteria	Phycisphaerae	Tepidisphaerales	Tepidisphaeraceae	Tepidisphaera	Tepidisphaera mucosa
ASV47	Planctomycetota	Planctomycetes	Planctomycetales	Planctomycetaceae	Calycomorphotria	Calycomorphotria hydatis
ASV49	Planctobacteria	Phycisphaerae	Phycisphaerales	Phycisphaeraceae	NA	NA
ASV50	Planctobacteria	Phycisphaerae	Tepidisphaerales	Tepidisphaeraceae	Tepidisphaera	Tepidisphaera mucosa
ASV51	Planctobacteria	Phycisphaerae	Tepidisphaerales	Tepidisphaeraceae	Tepidisphaera	Tepidisphaera mucosa
ASV52	Planctobacteria	Planctomycetes	Planctomycetales	Planctomycetaceae	Planctomyces	Planctomyces maris
ASV53	Planctobacteria	Phycisphaerae	Tepidisphaerales	Tepidisphaeraceae	NA	NA
ASV54	Patescibacteria	Saccharimonadia	Saccharimonadales	NA	NA	NA
ASV56	Planctobacteria	Planctomycetes	Pirellulales	Pirellulaceae	Pirellula	Pirellula sp.
ASV57	Planctobacteria	Phycisphaerae	Tepidisphaerales	Tepidisphaeraceae	Tepidisphaera	Tepidisphaera mucosa
ASV59	Planctobacteria	Phycisphaerae	Tepidisphaerales	Tepidisphaeraceae	Tepidisphaera	Tepidisphaera mucosa
ASV60	Planctobacteria	Planctomycetes	Gemmatales	Gemmataceae	Fimbriiglobus	Fimbriiglobus sp.
ASV61	Patescibacteria	Parcubacteria	Candidatus_Kaiserbacteria	NA	Candidatus Kaiserbacteria	Candidatus Kaiserbacteria
ASV62	Planctobacteria	Planctomycetes	Isosphaerales	Isosphaeraceae	Isosphaera	Isosphaera sp.
ASV63	Patescibacteria	Parcubacteria	NA	NA	NA	NA
ASV64	Planctobacteria	Planctomycetes	Planctomycetales	Planctomycetaceae	Planctomyces	Planctomyces maris

ASV68	Planctobacteria	Planctomycetes	Gemmatales	Gemmataceae	Gemmata	Gemmata sp.
ASV69	Planctobacteria	Phycisphaerae	Tepidisphaerales	Tepidisphaeraceae	Tepidisphaera	Tepidisphaera mucosa
ASV71	Planctobacteria	Phycisphaerae	Tepidisphaerales	Tepidisphaeraceae	Tepidisphaera	Tepidisphaera mucosa
ASV74	Planctobacteria	Phycisphaerae	Tepidisphaerales	Tepidisphaeraceae	Tepidisphaera	Tepidisphaera mucosa
ASV76	Planctobacteria	Planctomycetes	Isosphaerales	Isosphaeraceae	Aquisphaera	Aquisphaera sp.
ASV77	Planctobacteria	Planctomycetes	Pirellulales	Pirellulaceae	NA	NA
ASV78	Planctobacteria	Phycisphaerae	Tepidisphaerales	Tepidisphaeraceae	Tepidisphaera	Tepidisphaera mucosa
ASV80	Planctobacteria	Phycisphaerae	Tepidisphaerales	Tepidisphaeraceae	NA	NA
ASV81	Planctobacteria	Planctomycetes	Isosphaerales	Isosphaeraceae	Isosphaera	Isosphaera sp.
ASV83	Planctobacteria	Phycisphaerae	Tepidisphaerales	Tepidisphaeraceae	Tepidisphaera	Tepidisphaera mucosa
ASV84	Planctobacteria	Planctomycetes	Gemmatales	Gemmataceae	Gemmata	Gemmata sp.
ASV86	Planctobacteria	Phycisphaerae	Tepidisphaerales	Tepidisphaeraceae	Tepidisphaera	Tepidisphaera mucosa
ASV87	Planctobacteria	Phycisphaerae	Tepidisphaerales	Tepidisphaeraceae	Tepidisphaera	Tepidisphaera mucosa
ASV90	Planctobacteria	Planctomycetes	Gemmatales	Gemmataceae	Gemmata	Gemmata sp.
ASV92	Planctobacteria	Planctomycetes	Gemmatales	Gemmataceae	Fimbriiglobus	Fimbriiglobus sp.
ASV93	Planctobacteria	Phycisphaerae	Tepidisphaerales	Tepidisphaeraceae	NA	NA
ASV94	Planctobacteria	Planctomycetes	Isosphaerales	Isosphaeraceae	Isosphaera	Isosphaera sp.
ASV98	Planctobacteria	Planctomycetes	Gemmatales	Gemmataceae	Zavarzinella	Zavarzinella formosa
ASV100	Planctobacteria	Planctomycetes	Planctomycetales	Planctomycetaceae	Planctomyces	Planctomyces maris
ASV101	Planctobacteria	Phycisphaerae	Tepidisphaerales	Tepidisphaeraceae	NA	NA
ASV102	Planctobacteria	Phycisphaerae	Tepidisphaerales	Tepidisphaeraceae	NA	NA
ASV104	Verrucomicrobiota	Verrucomicrobiae	Verrucomicrobiales	Verrucomicrobiaceae		Roseimicrobium gellanilyticum
ASV106	Planctobacteria	Planctomycetes	Gemmatales	Gemmataceae	Gemmata	Gemmata sp.
ASV111	Planctobacteria	Phycisphaerae	Tepidisphaerales	Tepidisphaeraceae	NA	NA
ASV112	Planctobacteria	Phycisphaerae	Tepidisphaerales	Tepidisphaeraceae	NA	NA
ASV121	Planctobacteria	Planctomycetes	Gemmatales	Gemmataceae	Planctomicrobium	Planctomicrobium piriforme

ASV124	Planctobacteria	Phycisphaerae	Tepidisphaerales	Tepidisphaeraceae	Tepidisphaera	Tepidisphaera mucosa
ASV125	Planctobacteria	Planctomycetes	Gemmatales	Gemmataceae	Gemmata	Gemmata sp.
ASV126	Planctobacteria	Planctomycetes	Gemmatales	Gemmataceae	Gemmata	Gemmata sp.
ASV132	Planctobacteria	Phycisphaerae	Tepidisphaerales	Tepidisphaeraceae	Tepidisphaera	Tepidisphaera mucosa
ASV136	Planctobacteria	Phycisphaerae	Tepidisphaerales	Tepidisphaeraceae	NA	NA
ASV142	Patescibacteria	Parcubacteria	Candidatus_Adlerbacteria	NA	Candidatus	Candidatus Adlerbacteria
ASV143	Planctobacteria	Planctomycetes	Isosphaerales	Isosphaeraceae	Isosphaera	Isosphaera sp.
ASV144	Planctobacteria	Phycisphaerae	Tepidisphaerales	Tepidisphaeraceae	NA	NA
ASV152	Planctobacteria	Planctomycetes	Gemmatales	Gemmataceae	Gemmata	Gemmata sp.
ASV155	Planctobacteria	Planctomycetes	Planctomycetales	Planctomycetaceae	Caulifigura	Caulifigura coniformis
ASV159	Planctobacteria	Planctomycetes	Gemmatales	Gemmataceae	Fimbriiglobus	Fimbriiglobus sp.
ASV166	Planctobacteria	Planctomycetes	Isosphaerales	Isosphaeraceae	Isosphaera	Isosphaera sp.
ASV167	Planctobacteria	Phycisphaerae	Tepidisphaerales	Tepidisphaeraceae	NA	NA
ASV184	Planctobacteria	Phycisphaerae	Tepidisphaerales	Tepidisphaeraceae	Tepidisphaera	Tepidisphaera mucosa
ASV214	Planctobacteria	Phycisphaerae	Tepidisphaerales	Tepidisphaeraceae	Tepidisphaera	Tepidisphaera mucosa
ASV227	Planctobacteria	Phycisphaerae	Tepidisphaerales	Tepidisphaeraceae	NA	NA
ASV231	Planctobacteria	Planctomycetes	Pirellulales	Pirellulaceae	Pirellula	Pirellula sp.
ASV249	Planctobacteria	Phycisphaerae	Tepidisphaerales	CPla-3_termite_group	NA	NA
ASV253	Planctobacteria	Phycisphaerae	Tepidisphaerales	Tepidisphaeraceae	NA	NA
ASV333	Planctobacteria	Planctomycetes	Pirellulales	Pirellulaceae	Pirellula	Pirellula sp.
ASV506	Planctobacteria	Phycisphaerae	Tepidisphaerales	CPla-3_termite_group	NA	NA

Table S8. Bacterial eco-microbiota in soybean rhizosphere.

ASVs	Phylum	Class	Order	Family	Genus	Species
ASV1	Planctobacteria	Phycisphaerae	Tepidisphaerales	Tepidisphaeraceae	Tepidisphaera	Tepidisphaera mucosa
ASV2	Planctobacteria	Phycisphaerae	Tepidisphaerales	Tepidisphaeraceae	Tepidisphaera	Tepidisphaera mucosa
ASV6	Planctobacteria	Phycisphaerae	Tepidisphaerales	Tepidisphaeraceae	Tepidisphaera	Tepidisphaera mucosa

ASV7	Planctobacteria	Planctomycetes	Isosphaerales	Isosphaeraceae	Isosphaera	Isosphaera sp.
ASV15	Planctobacteria	Phycisphaerae	Phycisphaerales	Phycisphaeraceae	Algisphaera	Algisphaera agarilytica
ASV21	Planctobacteria	Planctomycetes	Pirellulales	Pirellulaceae	Lacipirellula	Lacipirellula parvula
ASV23	Planctobacteria	Planctomycetes	Gemmatales	Gemmataceae	Gemmata	Gemmata sp.
ASV26	Planctobacteria	Planctomycetes	Gemmatales	Gemmataceae	Gemmata	Gemmata sp.
ASV30	Planctobacteria	Phycisphaerae	Tepidisphaerales	Tepidisphaeraceae	Tepidisphaera	Tepidisphaera mucosa
ASV33	Planctobacteria	Phycisphaerae	Tepidisphaerales	Tepidisphaeraceae	Tepidisphaera	Tepidisphaera mucosa
ASV34	Planctobacteria	Phycisphaerae	Tepidisphaerales	Tepidisphaeraceae	Tepidisphaera	Tepidisphaera mucosa
ASV35	Planctobacteria	Phycisphaerae	Tepidisphaerales	Tepidisphaeraceae	Tepidisphaera	Tepidisphaera mucosa
ASV36	Planctobacteria	Planctomycetes	Gemmatales	Gemmataceae	Gemmata	Gemmata sp.
ASV37	Planctobacteria	Planctomycetes	Gemmatales	Gemmataceae	Gemmata	Gemmata sp.
ASV38	Planctobacteria	Planctomycetes	Gemmatales	Gemmataceae	Gemmata	Gemmata sp.
ASV40	Planctobacteria	Planctomycetes	Gemmatales	Gemmataceae	Gemmata	Gemmata sp.
ASV44	Proteobacteria	Betaproteobacteria	Burkholderiales	Burkholderiaceae	Ralstonia	Ralstonia solanacearum
ASV45	Planctobacteria	Phycisphaerae	Tepidisphaerales	Tepidisphaeraceae	Tepidisphaera	Tepidisphaera mucosa
ASV47	Planctomycetota	Planctomycetes	Planctomycetales	Planctomycetaceae	Calycomorphotria	Calycomorphotria hydatis
ASV48	Planctobacteria	Planctomycetes	Pirellulales	Pirellulaceae	NA	NA
ASV50	Planctobacteria	Phycisphaerae	Tepidisphaerales	Tepidisphaeraceae	Tepidisphaera	Tepidisphaera mucosa
ASV51	Planctobacteria	Phycisphaerae	Tepidisphaerales	Tepidisphaeraceae	Tepidisphaera	Tepidisphaera mucosa
ASV52	Planctobacteria	Planctomycetes	Planctomycetales	Planctomycetaceae	Planctomyces	Planctomyces maris
ASV56	Planctobacteria	Planctomycetes	Pirellulales	Pirellulaceae	Pirellula	Pirellula sp.
ASV59	Planctobacteria	Phycisphaerae	Tepidisphaerales	Tepidisphaeraceae	Tepidisphaera	Tepidisphaera mucosa
ASV64	Planctobacteria	Planctomycetes	Planctomycetales	Planctomycetaceae	Planctomyces	Planctomyces maris
ASV65	Planctobacteria	Planctomycetes	Pirellulales	Pirellulaceae	Pirellula	Pirellula sp.
ASV66	Chloroflexi	Chloroflexia	Kallotenuales	Kallotenuaceae	Kallotenue	Kallotenue papyrolyticum
ASV75	Planctobacteria	Planctomycetes	Gemmatales	Gemmataceae	Gemmata	Gemmata sp.
ASV78	Planctobacteria	Phycisphaerae	Tepidisphaerales	Tepidisphaeraceae	Tepidisphaera	Tepidisphaera mucosa
ASV79	Chloroflexi	Chloroflexia	Kallotenuales	Kallotenuaceae	Kallotenue	Kallotenue papyrolyticum
ASV81	Planctobacteria	Planctomycetes	Isosphaerales	Isosphaeraceae	Isosphaera	Isosphaera sp.

ASV82	Planctobacteria	Planctomycetes	Gemmatales	Gemmataceae	Fimbriiglobus	Fimbriiglobus ruber
ASV83	Planctobacteria	Phycisphaerae	Tepidisphaerales	Tepidisphaeraceae	Tepidisphaera	Tepidisphaera mucosa
ASV84	Planctobacteria	Planctomycetes	Gemmatales	Gemmataceae	Gemmata	Gemmata sp.
ASV85	Planctobacteria	Planctomycetes	Gemmatales	Gemmataceae	Gemmata	Gemmata sp.
ASV88	Planctobacteria	Planctomycetes	Gemmatales	Gemmataceae	Zavarzinella	Zavarzinella formosa
ASV89	Planctobacteria	Planctomycetes	Gemmatales	Gemmataceae	Gemmata	Gemmata sp.
ASV93	Planctobacteria	Phycisphaerae	Tepidisphaerales	Tepidisphaeraceae	NA	NA
ASV94	Planctobacteria	Planctomycetes	Isosphaerales	Isosphaeraceae	Isosphaera	Isosphaera sp.
ASV96	Planctobacteria	Planctomycetes	Gemmatales	Gemmataceae	Zavarzinella	Zavarzinella formosa
ASV97	Planctobacteria	Planctomycetes	Gemmatales	Gemmataceae	Gemmata	Gemmata sp.
ASV100	Planctobacteria	Planctomycetes	Planctomycetales	Planctomycetaceae	Planctomyces	Planctomyces maris
ASV103	Planctobacteria	Planctomycetes	Pirellulales	Pirellulaceae	Pirellula	Pirellula sp.
ASV105	Planctobacteria	Planctomycetes	Gemmatales	Gemmataceae	Gemmata	Gemmata sp.
ASV108	Planctobacteria	Planctomycetes	Gemmatales	Gemmataceae	Gemmata	Gemmata sp.
ASV110	Planctobacteria	Phycisphaerae	Tepidisphaerales	Tepidisphaeraceae	Tepidisphaera	Tepidisphaera mucosa
ASV111	Planctobacteria	Phycisphaerae	Tepidisphaerales	Tepidisphaeraceae	NA	NA
ASV114	Planctobacteria	Planctomycetes	Planctomycetales	Rubinisphaeraceae	Caulifigura	Caulifigura coniformis
ASV115	Planctobacteria	Planctomycetes	Gemmatales	Gemmataceae	Gemmata	Gemmata sp.
ASV117	Planctobacteria	Planctomycetes	Gemmatales	Gemmataceae	Gemmata	Gemmata sp.
ASV119	Planctobacteria	Planctomycetes	Isosphaerales	Isosphaeraceae	Paludisphaera	Paludisphaera borealis
ASV121	Planctobacteria	Planctomycetes	Gemmatales	Gemmataceae	Planctomicrobium	Planctomicrobium piriforme
ASV123	Planctobacteria	Planctomycetes	Gemmatales	Gemmataceae	Gemmata	Gemmata sp.
ASV124	Planctobacteria	Phycisphaerae	Tepidisphaerales	Tepidisphaeraceae	Tepidisphaera	Tepidisphaera mucosa
ASV128	Planctobacteria	Planctomycetes	Gemmatales	Gemmataceae	Gemmata	Gemmata sp.
ASV132	Planctobacteria	Phycisphaerae	Tepidisphaerales	Tepidisphaeraceae	Tepidisphaera	Tepidisphaera mucosa
ASV133	Planctobacteria	Planctomycetes	Gemmatales	Gemmataceae	Gemmata	Gemmata sp.
ASV135	Planctobacteria	Planctomycetes	Pirellulales	Pirellulaceae	Thermostilla	Thermostilla marina
ASV137	Planctobacteria	Planctomycetes	Pirellulales	Pirellulaceae	Pirellula	Pirellula sp.
ASV139	Planctobacteria	Planctomycetes	Pirellulales	Pirellulaceae	Pirellula	Pirellula sp.

ASV140	Planctobacteria	Phycisphaerae	Tepidisphaerales	Tepidisphaeraceae	NA	NA
ASV144	Planctobacteria	Phycisphaerae	Tepidisphaerales	Tepidisphaeraceae	NA	NA
ASV145	Planctobacteria	Planctomycetes	Gemmatales	Gemmataceae	Gemmata	Gemmata sp.
ASV150	Proteobacteria	Betaproteobacteria	Burkholderiales	Burkholderiaceae	Ralstonia	Ralstonia solanacearum
ASV153	Planctobacteria	Planctomycetes	Pirellulales	Pirellulaceae	Pirellula	Pirellula sp.
ASV154	Planctobacteria	Planctomycetes	Gemmatales	Gemmataceae	Gemmata	Gemmata sp.
ASV163	Planctobacteria	Planctomycetes	Gemmatales	Gemmataceae	Gemmata	Gemmata sp.
ASV165	Planctobacteria	Planctomycetes	Gemmatales	Gemmataceae	Limnoglobus	Limnoglobus roseus
ASV168	Planctobacteria	Planctomycetes	Pirellulales	Pirellulaceae	Thermostilla	Thermostilla marina
ASV169	Planctobacteria	Planctomycetes	Gemmatales	Gemmataceae	Gemmata	Gemmata sp.
ASV170	Planctobacteria	Planctomycetes	Pirellulales	Pirellulaceae	Pirellula	Pirellula sp.
ASV172	Planctobacteria	Phycisphaerae	Tepidisphaerales	Tepidisphaeraceae	NA	NA
ASV176	Chloroflexi	Chloroflexia	Kallotenuales	Kallotenuaceae	Kallotenue	Kallotenue papyrolyticum
ASV180	Verrucomicrobiota	Verrucomicrobiae	Chthoniobacterales	Chthoniobacteraceae	Candidatus_Udaeobacter	Candidatus_Udaeobacter
ASV183	Planctobacteria	Planctomycetes	Pirellulales	Pirellulaceae	NA	NA
ASV184	Planctobacteria	Phycisphaerae	Tepidisphaerales	Tepidisphaeraceae	Tepidisphaera	Tepidisphaera mucosa
ASV185	Planctobacteria	Planctomycetes	Pirellulales	Pirellulaceae	Candidatus	Candidatus Anammoximicrobium moscowii
ASV187	Planctobacteria	Planctomycetes	Pirellulales	Pirellulaceae	Pirellula	Pirellula sp.
ASV197	Planctobacteria	Planctomycetes	Pirellulales	Pirellulaceae	Pirellula	Pirellula sp.
ASV199	Planctobacteria	Planctomycetes	Gemmatales	Gemmataceae	Gemmata	Gemmata sp.
ASV200	Planctobacteria	Planctomycetes	Gemmatales	Gemmataceae	Zavarzinella	Zavarzinella formosa
ASV203	Planctobacteria	Planctomycetes	Gemmatales	Gemmataceae	Gemmata	Gemmata sp.
ASV208	Planctobacteria	Phycisphaerae	Tepidisphaerales	Tepidisphaeraceae	NA	NA
ASV209	Planctobacteria	Phycisphaerae	Tepidisphaerales	Tepidisphaeraceae	NA	NA
ASV210	Planctobacteria	Planctomycetes	Pirellulales	Pirellulaceae	Pirellula	Pirellula sp.
ASV213	Planctobacteria	Planctomycetes	Gemmatales	Gemmataceae	Fimbriiglobus	Fimbriiglobus ruber
ASV221	Planctobacteria	Planctomycetes	Gemmatales	Gemmataceae	Gemmata	Gemmata sp.
ASV223	Planctobacteria	Planctomycetes	Gemmatales	Gemmataceae	Gemmata	Gemmata sp.
ASV224	Chloroflexi	Chloroflexia	Kallotenuales	Kallotenuaceae	Kallotenue	Kallotenue papyrolyticum

ASV227	Planctobacteria	Phycisphaerae	Tepidisphaerales	Tepidisphaeraceae	NA	NA
ASV228	Planctobacteria	Planctomycetes	Pirellulales	Pirellulaceae	Lacipirellula	Lacipirellula parvula
ASV229	Planctobacteria	Planctomycetes	Gemmatales	Gemmataceae	Gemmata	Gemmata sp.
ASV230	Planctobacteria	Planctomycetes	Planctomycetales	Planctomycetaceae	Planctomyces	Planctomyces maris
ASV232	Planctobacteria	Planctomycetes	Isosphaerales	Isosphaeraceae	Aquisphaera	Aquisphaera giovannonii
ASV234	Planctobacteria	Planctomycetes	Gemmatales	Gemmataceae	Zavarzinella	Zavarzinella formosa
ASV239	Planctobacteria	Planctomycetes	Pirellulales	Pirellulaceae	Lacipirellula	Lacipirellula limnantheis
ASV244	Planctobacteria	Planctomycetes	Gemmatales	Gemmataceae	Zavarzinella	Zavarzinella formosa
ASV250	Planctobacteria	Planctomycetes	Pirellulales	Pirellulaceae	Pirellula	Pirellula sp.
ASV251	Planctobacteria	Planctomycetes	Gemmatales	Gemmataceae	Gemmata	Gemmata sp.
ASV258	Planctobacteria	Planctomycetes	Gemmatales	Gemmataceae	Gemmata	Gemmata sp.
ASV264	Planctobacteria	Planctomycetes	Gemmatales	Gemmataceae	Gemmata	Gemmata sp.
ASV278	Planctobacteria	Planctomycetes	Pirellulales	Pirellulaceae	Pirellula	Pirellula sp.
ASV284	Planctobacteria	Planctomycetes	Pirellulales	Pirellulaceae	Thermostilla	Thermostilla marina
ASV298	Planctobacteria	Planctomycetes	Pirellulales	Pirellulaceae	Pirellula	Pirellula sp.
ASV302	Planctobacteria	Planctomycetes	Pirellulales	Pirellulaceae	Lacipirellula	Lacipirellula limnantheis
ASV303	Planctobacteria	Planctomycetes	Gemmatales	Gemmataceae	Gemmata	Gemmata sp.
ASV349	Planctobacteria	Planctomycetes	Pirellulales	Pirellulaceae	Pirellula	Pirellula sp.
ASV353	Planctobacteria	Planctomycetes	Planctomycetales	Planctomycetaceae	Planctomyces	Planctomyces maris
ASV360	Planctobacteria	Planctomycetes	Pirellulales	Pirellulaceae	Pirellula	Pirellula sp.
ASV376	Planctobacteria	Planctomycetes	Gemmatales	Gemmataceae	Gemmata	Gemmata sp.
ASV392	Chloroflexi	Betaproteobacteria	Burkholderiales	Burkholderiaceae	Ralstonia	Ralstonia solanacearum
ASV429	Planctobacteria	Planctomycetes	Gemmatales	Gemmataceae	Fimbriiglobus	Fimbriiglobus ruber
ASV598	Planctobacteria	Planctomycetes	Pirellulales	Pirellulaceae	Pirellula	Pirellula sp.
ASV679	Planctobacteria	Planctomycetes	Pirellulales	Pirellulaceae	Pirellula	Pirellula sp.

Table S9. Eco-mycobiota in soybean root and rhizosphere.

A. Eco-mycobiota in soybean root.

ASV	Phylum	Class	Order	Family	Genus	Species	total abundance	relative abundance

ASV2	Ascomycota	Sordariomycetes	Sordariales	Chaetomiaceae	Humicola	Humicola fuscoatra	25205	0.01255245

B. Eco-mycobiota in soybean rhizosphere.

ASV	Phylum	Class	Order	Family	Genus	Species	total abundance	relative abundance
ASV2	Ascomycota	Sordariomycetes	Sordariales	Chaetomiaceae	Humicola	Humicola fuscoatra	490161	0.24410712
ASV3	Ascomycota	Sordariomycetes	Sordariales	Chaetomiaceae	Humicola	Humicola fuscoatra	97659	0.04863557
ASV7	Ascomycota	Sordariomycetes	Glomerellales	Plectosphaerellaceae	Gibellulopsis	Gibellulopsis sp.	57385	0.02857854
ASV8	Ascomycota	Sordariomycetes	Sordariales	Chaetomiaceae	Chaetomium	Chaetomium longiciliata	35287	0.01757343
ASV9	Ascomycota	Pezizomycetes	Pezizales	Pyronemataceae	Pseudombrophila	Pseudombrophila subalpina	43577	0.02170196
ASV10	Ascomycota	Sordariomycetes	Sordariales	Sordariaceae	Sordaria	Sordaria tomentoalba	43452	0.02163971
ASV11	Ascomycota	Sordariomycetes	Sordariales	Chaetomiaceae	Cladorrhinum	Cladorrhinum sp.	34473	0.01716804
ASV16	Ascomycota	Sordariomycetes	Sordariales	Chaetomiaceae	Chaetomium	Chaetomium sp.	24180	0.01204198
ASV17	Ascomycota	Sordariomycetes	NA	NA	NA	Sordariomycetes sp.	20858	0.01038758
ASV20	Ascomycota	Sordariomycetes	Sordariales	Sordariaceae	Sordaria	Sordaria sp.	18048	0.00898816
ASV23	Ascomycota	Sordariomycetes	Sordariales	Chaetomiaceae	Staphylotrichum	Staphylotrichum coccosporum	14438	0.00719033
ASV26	Ascomycota	Sordariomycetes	Hypocreales	Stachybotryaceae	Myrothecium	Myrothecium sp.	13676	0.00681084
ASV28	Ascomycota	Sordariomycetes	Sordariales	Chaetomiaceae	NA	Chaetomiaceae sp.	12312	0.00613155
ASV32	Ascomycota	Leotiomycetes	NA	Pseudeurotiaceae	Pseudeurotium	Pseudeurotium hygrophilum	9883	0.00492187
ASV57	Ascomycota	Sordariomycetes	Sordariales	Chaetomiaceae	Podospora	Podospora multipilosa	203	0.0001011

Table S10. Global hub taxa identified in soybean microbiome.

ASV	Phylum	Class	Order	Family	Genus	Species
BASV6	Planctobacteria	Phycisphaerae	Tepidisphaerales	Tepidisphaeraceae	Tepidisphaera	Tepidisphaera mucosa
BASV14	Planctobacteria	Phycisphaerae	Tepidisphaerales	Tepidisphaeraceae	Tepidisphaera	Tepidisphaera mucosa
BASV16	Planctobacteria	Phycisphaerae	Tepidisphaerales	Tepidisphaeraceae	Tepidisphaera	Tepidisphaera mucosa
BASV30	Planctobacteria	Phycisphaerae	Tepidisphaerales	Tepidisphaeraceae	Tepidisphaera	Tepidisphaera mucosa
BASV35	Planctobacteria	Phycisphaerae	Tepidisphaerales	Tepidisphaeraceae	Tepidisphaera	Tepidisphaera mucosa
BASV44	Proteobacteria	Betaproteobacteria	Burkholderiales	Burkholderiaceae	Ralstonia	Ralstonia solanacearum
BASV57	Planctobacteria	Phycisphaerae	Tepidisphaerales	Tepidisphaeraceae	Tepidisphaera	Tepidisphaera mucosa
BASV58	Planctobacteria	Planctomycetes	Gemmatales	Gemmataceae	Gemmata	Gemmata sp.

BASV87	Planctobacteria	Phycisphaerae	Tepidisphaerales	Tepidisphaeraceae	Tepidisphaera	Tepidisphaera mucosa
BASV91	Planctobacteria	Phycisphaerae	Tepidisphaerales	Tepidisphaeraceae	Tepidisphaera	Tepidisphaera mucosa
BASV132	Planctobacteria	Phycisphaerae	Tepidisphaerales	Tepidisphaeraceae	Tepidisphaera	Tepidisphaera mucosa
BASV148	Chloroflexi	Chloroflexia	Chloroflexales	Chloroflexaceae	Chloroflexus	Chloroflexus aurantiacus
BASV175	Planctobacteria	Planctomycetes	Pirellulales	Pirellulaceae	Thermostilla	Thermostilla marina
BASV196	Planctobacteria	Phycisphaerae	Tepidisphaerales	Tepidisphaeraceae	Tepidisphaera	Tepidisphaera mucosa
BASV200	Planctobacteria	Planctomycetes	Gemmatales	Gemmataceae	Zavarzinella	Zavarzinella formosa
BASV268	Planctobacteria	Planctomycetes	Gemmatales	Gemmataceae	Gemmata	Gemmata sp.
BASV308	Planctobacteria	Phycisphaerae	Tepidisphaerales	Tepidisphaeraceae	Tepidisphaera	Tepidisphaera mucosa
BASV311	Planctobacteria	Planctomycetes	Gemmatales	Gemmataceae	Gemmata	Gemmata sp.
BASV577	Planctobacteria	Planctomycetes	Pirellulales	Pirellulaceae	Pirellula	Pirellula sp.
